# Retraction notice to "Wireless sensor network-based machine learning framework for smart cities in intelligent waste management" [Heliyon 10 (2024) e36271]

**DOI:** 10.1016/j.heliyon.2025.e43552

**Published:** 2025-06-24

**Authors:** Karan Belsare, Manwinder Singh, Anudeep Gandam, Varakumari Samudrala, Rajesh Singh, Naglaa F. Soliman, Sudipta Das, Abeer D. Algarni

**Affiliations:** a School of Electronics and Electrical Engineering, Lovely Professional University, Phagwara, Punjab, 144411, India; b School of Computer Science and Engineering, Lovely Professional University, Phagwara, Punjab, 144411, India; c Department of Electronics and Communication Engineering, NRI Institute of Technology, Agiripalli, Vijayawada, Andhra Pradesh, 521212, India; d Division of Research and Innovation, Uttaranchal University, Dehradun, Uttrakhand, 248007, India; e Department of Information Technology, College of Computer and Information Sciences, Princess Nourah bint Abdulrahman University, P.O. Box 84428, Riyadh, 11671, Saudi Arabia; f Department of Electronics and Communication Engineering, IMPS College of Engineering and Technology, Malda, 732103, West Bengal, India

This article has been retracted: please see Elsevier policy on article withdrawal (https://www.elsevier.com/about/policies-and-standards/article-withdrawal).

This article has been retracted at the request of the Editor.

An investigation conducted on behalf of the journal by Elsevier's Research Integrity & Publishing Ethics team found phrases that make some passages in the article difficult to parse. The authors were requested to explain the use of these passages of text but were unable to do so.

Furthermore, the Elsevier's Research Integrity & Publishing Ethics team on behalf of the journal identified references that are irrelevant to the article. The authors were asked to comment upon the presence of these references in their work but were unable to satisfactorily address the reason for the references.

Consequently, the editor no longer has confidence in the integrity and the findings of the article and has decided to retract it. The scientific community takes a very strong view on this matter and apologies are offered to readers of the journal that this was not detected during the submission process.

The authors disagree with retraction and dispute the grounds for it.

